# Resolvin RvD2 reduces hypothalamic inflammation and rescues mice from diet-induced obesity

**DOI:** 10.1186/s12974-016-0777-2

**Published:** 2017-01-05

**Authors:** Livia B. Pascoal, Bruna Bombassaro, Albina F. Ramalho, Andressa Coope, Rodrigo F. Moura, Felipe Correa-da-Silva, Leticia Ignacio-Souza, Daniela Razolli, Diogo de Oliveira, Rodrigo Catharino, Licio A. Velloso

**Affiliations:** 1 Obesity and Comorbidities Research Center, Laboratory of Cell Signaling, University of Campinas, Campinas, SP 13084-761 Brazil; 2 Faculty of Pharmaceutical Sciences, University of Campinas, Campinas, Brazil

**Keywords:** Hypothalamus, Nutrient, Metabolism, Brain, Lipid

## Abstract

**Background:**

Diet-induced hypothalamic inflammation is an important mechanism leading to dysfunction of neurons involved in controlling body mass. Studies have shown that polyunsaturated fats can reduce hypothalamic inflammation. Here, we evaluated the presence and function of RvD2, a resolvin produced from docosahexaenoic acid, in the hypothalamus of mice.

**Methods:**

Male Swiss mice were fed either chow or a high-fat diet. RvD2 receptor and synthetic enzymes were evaluated by real-time PCR and immunofluorescence. RvD2 was determined by mass spectrometry. Dietary and pharmacological approaches were used to modulate the RvD2 system in the hypothalamus, and metabolic phenotype consequences were determined.

**Results:**

All enzymes involved in the synthesis of RvD2 were detected in the hypothalamus and were modulated in response to the consumption of dietary saturated fats, leading to a reduction of hypothalamic RvD2. GPR18, the receptor for RvD2, which was detected in POMC and NPY neurons, was also modulated by dietary fats. The substitution of saturated by polyunsaturated fats in the diet resulted in increased hypothalamic RvD2, which was accompanied by reduced body mass and improved glucose tolerance. The intracerebroventricular treatment with docosahexaenoic acid resulted in increased expression of the RvD2 synthetic enzymes, increased expression of anti-inflammatory cytokines and improved metabolic phenotype. Finally, intracerebroventricular treatment with RvD2 resulted in reduced adiposity, improved glucose tolerance and increased hypothalamic expression of anti-inflammatory cytokines.

**Conclusions:**

Thus, RvD2 is produced in the hypothalamus, and its receptor and synthetic enzymes are modulated by dietary fats. The improved metabolic outcomes of RvD2 make this substance an attractive approach to treat obesity.

**Electronic supplementary material:**

The online version of this article (doi:10.1186/s12974-016-0777-2) contains supplementary material, which is available to authorized users.

## Background

The prompt resolution of acute inflammation triggered by infectious agents, trauma or chemical stimulus plays an essential role in avoiding chronicity and unwanted tissue damage that could result from an unrestrained response to the original harmful stimulus [1]. Lipoxins [2], resolvins [3, 4] and protectins [5] are families of endogenously produced, lipid-derived substances that act in the resolution phase of acute inflammatory processes [6]. A number of recent studies have characterised the mechanisms involved in the synthesis and action of these substances in different inflammatory conditions, providing evidence for their essential role in tissue protection and demonstrating their potential use as therapeutic tools in several diseases [7–10].

Resolvin D2 (RvD2), one of the members of the resolvin family, is produced from the ω3-polyunsaturated fatty acid, docosahexaenoic acid (DHA), as a result of a series of reactions catalysed by lipoxygenases [3]. The anti-inflammatory and pro-resolution effects of RvD2 are mediated, at least in part, by the pertussis-sensitive G-protein-coupled receptor (GPCR), GPR18, by a signalling mechanism yet to be fully elucidated [10, 11]. Although most studies have explored the role of resolvins in acute inflammatory conditions, a recent study has provided evidence that both RvD2 and resolvin D1 (RvD1) can modulate the chronic inflammatory process that takes place in the adipose tissue of obese subjects [12]. In addition, treatment with 17-hydroxydocosahexaenoic acid (17-HDHA), a precursor of RvD2, reduced inflammation and corrected systemic insulin resistance in obese diabetic rodents [13].

Currently, obesity is one of the most prevalent diseases in the world (http://www.who.int/mediacentre/factsheets/fs311/en/). It is the main risk factor for type 2 diabetes mellitus (T2D) and is also an important predisposing condition for hypertension, atherosclerosis and some types of cancer [14]. Saturated fatty acids present in the diet induce an inflammatory response in the hypothalamus, leading to a dysfunctional regulation of caloric intake and energy expenditure [15–19], which plays an important role in the genesis and perpetuation of obesity [20, 21]. In fact, a number of pharmacological and genetic approaches used to dampen obesity-linked hypothalamic inflammation result in the reversal of the obese phenotype in animal models [16–18, 20, 22]. Recent studies have also shown that increased content of ω3 fatty acids in the diet or direct hypothalamic injection of ω3 fatty acids can reduce obesity-linked hypothalamic inflammation, increase POMC neuron-specific neurogenesis and attenuate the obese phenotype [23, 24].

Because ω3 fatty acids are precursors of RvD2, we evaluated the activity of this system in the hypothalamus of obese rodents. Here, we demonstrate that consumption of a diet rich in saturated fatty acids reduces the amount of RvD2 in the hypothalamus, while dietary supplementation or direct hypothalamic injection of ω3 fatty acids stimulates its synthesis. Administration of exogenous RvD2 reduces diet-induced hypothalamic inflammation and rescues from the obese phenotype. Thus, direct or indirect methods leading to the increase of RvD2 in the hypothalamus are potentially useful approaches to attenuate hypothalamic inflammation and dysfunction in obesity.

## Methods

### Chemicals and reagents

All of the reagents for SDS-polyacrylamide gel electrophoresis and immunoblotting were from Bio-Rad (Richmond, CA, USA). HEPES, phenylmethylsulfonyl fluoride, aprotinin, dithiothreitol, Triton X-100, Tween 20, glycerol and BSA (fraction V) were purchased from Sigma Chemical Co. (St. Louis, MO, USA). The antibodies against GPR18 (sc79503), NPY (sc133080) and Iba1 (sc28530) were from Santa Cruz Biotechnology (Santa Cruz, CA, USA). The reagents for chemiluminescence protein labelling in immunoblots were purchased from Amersham (Aylesbury, UK). FITC-conjugated anti-rabbit (sc2012), FITC-conjugated anti-goat (sc2024), Cy3-conjugated goat anti-mouse (ab6946), Cy3-conjugated donkey anti-goat (ab6949), rhodamine-conjugated anti-rabbit (sc2091) and rhodamine-conjugated anti-goat (sc2094) antibodies were from Santa Cruz Biotechnology (Santa Cruz, CA, USA). The lipid mediator resolvin D2 (sc-351847A) was from Santa Cruz Biotechnology (Santa Cruz, CA, USA). Reagents for the real-time PCR analysis were from Invitrogen (Carlsbad, CA, USA) and Applied Biosystems (Foster City, CA, USA). TaqMan primers for PLA2 (Mm00448161_m1), 15-LOX (Mm00507789_m1), 5-LOX (Mm01182747_m1), GPR18 (Mm01224541_s1), TNFα (Mm00443258_m1), IL1β (Mm00434228_m1), IL6 (Mm00446190_m1), IL10 (Mm01288386_m1), PGC1α (Mm00447183_m1), UCP1 (Mm01244861_m1) and glyceraldehyde-3-phosphate dehydrogenase (GAPD) (#4352339E) were obtained from Applied Biosystems.

### Experimental animals

Male Swiss mice originally imported from Jackson Laboratory and currently bred at the University of Campinas Breeding Center were used in the study. The investigation was conducted in accordance with the principles and procedures described by the National Institutes of Health Guidelines for the Care and Use of Experimental Animals and was previously approved by the University of Campinas Ethical Committee (ID 2011/2341-1). The animals were maintained at 21 ± 3 °C, on a 12-h artificial light/dark cycle and housed in individual cages. By 5 weeks old, the mice were assigned in three groups, with the same body weight mean: standard rodent chow diet (CT), high-fat diet (HF; 60% of energy value from fat, Table 1) and high-fat diet supplemented with omega 3 (HFS; HF supplemented with 20% omega 3, Table 1) by the time specified in the protocol.

**Table 1:** Composition of the experimental diets

	CT (g)	HF (g)	HFS (g)
Starch	427.5	115.5	115.5
Casein	200	200	200
Dextrin	132	132	132
Saccharose	100	100	100
Soy oil	40	40	40
Lard	0	312	104
Flaxseed oil	0	0	208
Dietary fiber	50	50	50
Minerals	35	35	35
Vitamins	10	10	10
Cysteine	3	3	3
Choline	2.5	2.5	2.5
kcal/1000 g	3798	5358	5358

### Experimental protocols

For evaluation and characterisation of biosynthetic pathways of RvD2, generated enzymatically from DHA, mice were fed for 16 weeks on either the chow diet or the HF. By the end of this period, the hypothalamus was removed and RNA extracts were employed in the real-time PCR analysis. In another set of experiments, mice were assigned to the HF or chow diet for 16 weeks and another group of mice was assigned to the HF for 8 weeks following 8 weeks on a HF supplemented with 20% omega 3 (HFS). Food intake and body mass were measured during this period. At the 15th week, the animals were subjected to the intraperitoneal glucose tolerance test. Subsequently, the hypothalamus was removed and employed in the MALDI-MSI analysis (as described below) and real-time PCR analysis for the identification and characterisation of RvD2 in this experimental model. In order to determine the impact of direct intracerebroventricular (icv) injection of DHA on the RvD2 system in the hypothalamus, mice were fed a HF for 4 weeks and were subsequently stereotaxically instrumented in a Stoelting stereotaxic apparatus to receive a cannula placed in the lateral hypothalamic ventricle, using the following stereotaxic coordinates: anteroposterior 0.34 mm, lateral 1.0 mm and dorsoventral 2.2 mm. The correct position of the cannula was tested 5 days after surgery by evaluation of the thirst response elicited by intracerebroventricular angiotensin II (10^6^ M). After 1 week, icv-cannulated mice were treated once a day for 4 days with 2 μL of saline or 2 μL of DHA (5, 10 or 20 ng). In another experiment, mice were fed a HF for 8 weeks and were subsequently stereotaxically instrumented, as previously described in this section. After 1 week, the icv-cannulated mice were treated once a day for 11 days with 2 μL of saline or 2 μL of RvD2 (3 or 50 ng). The doses of RvD2 were defined based on a previous study [9]. Food intake and body mass were measured during the treatment period. On the seventh and tenth days of treatment, the animals were subjected to an intracerebroventricular leptin tolerance test and intraperitoneal glucose tolerance test, respectively. In each group, some mice were randomly selected for indirect calorimetry and spontaneous physical activity measurements. At the end of the experiment, the hypothalamus and brown adipose tissue were collected for real-time PCR analysis. Outlines of the different protocols are depicted in the figures that show the respective experiments.

### ipGTT and icvLTT

The intraperitoneal glucose tolerance test (ipGTT) and intracerebroventricular leptin tolerance test (icvLTT) were performed on food-deprived (6 h) non-anaesthetised mice. Blood glucose levels were measured using an OptiumTM mini (Abbott Diabetes Care, Alameda, CA, USA) handheld glucometer with appropriate test strips. For ipGTT, a solution of 20% glucose (2.0 g/kg body weight) was administered into the peritoneal cavity. Blood samples were collected from the tail vein at 30, 60, 90 and 120 min for determination of glucose concentrations. The area under the curve (AUC) was calculated using these values. For icvLTT, food intake was measured 2, 4, 6 and 12 h following icv injection of leptin (10^−6^ M). These values were used for determining leptin sensitivity.

### RNA extraction and quantitative real-time PCR

Total RNA was extracted using a commercially available acid-phenol reagent Trizol (Invitrogen Corp.). RNA concentration, purity and integrity were confirmed spectrophotometrically using a Nanodrop (ND-1000; Nanodrop Technologies, Wilmington, DE). The first-strand cDNA was synthesised using SuperScript III reverse transcriptase and random hexamer primers, as described in the manufacturer’s protocol (Invitrogen Corp.) The quantitative PCR was run to determine the expression of TNFα, IL1β, IL6, IL10, PLA2, 15-LOX, 5-LOX and GPR18 in the hypothalamus of mice and to determine the expression of PGC1α and UCP1 in the brown adipose tissue (BAT) of mice using primers supplied with commercially available assays from Applied Biosystems. The endogenous gene was GAPD (glyceraldehyde-3-phosphate dehydrogenase (Applied Biosystems)). A real-time PCR analysis of gene expression was carried out in an ABI Prism 7500 sequence detection system (Applied Biosystems). The optimal concentration of complementary DNA and primers and the maximum efficiency of amplification were obtained through a 5-point, two-fold dilution curve analysis for each gene. Amplification was performed in a 20-μL final volume containing 40–50 ng of reverse-transcribed RNA according to the manufacturer’s recommendations using the TaqMan PCR master mix. Real-time data were analysed using the Sequence Detector System 1.7 (Applied Biosystems). Results were expressed as the relative transcript amount, as previously optimised [25].

### Immunofluorescence staining

For the histological analysis, hypothalamic tissue samples were frozen sectioned and processed routinely for immunofluorescence staining. Coronal sections of the hypothalamus (5 μm) were double-labelled with anti-GPR18 antibodies and specific primary antibodies against markers related to the different cell types, including NPY, POMC and Iba1. Thereafter, the sections were incubated with specific FITC or rhodamine-conjugated IgG secondary antibodies. Immunofluorescence imaging was performed to evaluate the distribution of GPR18 in the mouse’s hypothalamus.

### MALDI-MSI analyses

Obtained brain tissue sections were set on MALDI-appropriate stainless steel plates (GMS-Thermo, CA, USA) and then coated with a 10 mg/mL (50% methanol to acetonitrile) solution of alpha-cyano-4-hydroxycinnamic acid (CHCA) matrix (Sigma Aldrich, PA, USA). For an even distribution, a customised system with a commercial airbrush was utilised to spray the matrix. The MALDI-LTQ-XL instrument (Thermo Fisher, San Jose, CA, USA) with a tissue imaging feature was utilised in the mass spectrometry data acquisition. Operating conditions were set as follows: 4 μJ laser power, 50 μm raster step size, standardised sample size of 600 × 600 μm focused on the coronal sections of the hypothalamus, three laser shots per step and 30 eV for the helium collision-induced dissociation in fragmentation reactions (MS/MS). A survey scan was performed in the mass-to-charge ratio (m/z) range of 50 to 500. Samples were analysed in the negative ion mode. Compound structures were proposed using MS/MS data supported by software calculations with Mass Frontier (v. 6.0, Thermo Scientific, CA, USA), as well as previous data from the literature [25]. Imaging data were run in replicates for all described animal conditions (chow, HF and HDFω3) and were processed using ImageQuest software (Thermo Scientific, San Jose, CA, USA). Relative quantification was performed using ImageJ (National Institutes of Health, USA–Open Source) on images in grey scale. Since the analysed areas were the same size (in pixel numbers) for all the replicates, ImageJ was able to assign a non-dimensional value for each sample image. This result is based on the intensity of each pixel and can be compared among all samples to determine the relative levels of the desired molecule.

### Indirect calorimetry and spontaneous physical activity

Oxygen consumption/carbon dioxide production and spontaneous physical activity were measured in the fed animals through a computer-controlled, open-circuit calorimeter system (LE405 gas analyser; Panlab-Harvard Apparatus). Mice were singly housed in clear respiratory chambers and room air was passed through chambers at a flow rate of ten times the body weight of each animal. The air-flow within each chamber was monitored using a sensor (Air Supply and Switching; Panlab-Harvard Apparatus). Gas sensors were calibrated prior to the onset of the experiments using primary gas standards containing known concentrations of O_2_, CO_2_ and N_2_ (liquid air). The analyses were performed over a 24-h period. Outdoor air reference values were sampled after every four measurements. Sample air was sequentially passed through O_2_ and CO_2_ sensors for the determination of O_2_ and CO_2_ content, from which the measures of oxygen consumption (VO_2_) and carbon dioxide production (VCO_2_) were estimated. VO_2_ and VCO_2_ were calculated by Metabolism version 2.2 software based on the Withers equation and are expressed in millilitres per hour per gram. The respiratory quotient was calculated as VCO_2_/VO_2_. Energy expenditure was estimated as VO_2_/body mass (grams) [26].

### Statistical analysis

All results are reported as mean ± SEM. Differences between the treatment groups were evaluated using an unpaired Student *t* test or a one-way analysis of variance (ANOVA). When the ANOVA indicated significance, a Tukey-Kramer post hoc test was performed (GraphPad Software, San Diego, CA). *p* < 0.05 was accepted as being statistically significant.

## Results

### GPR18 is widely expressed in the neurons of the medium-basal hypothalamus

**Fig. 1:**
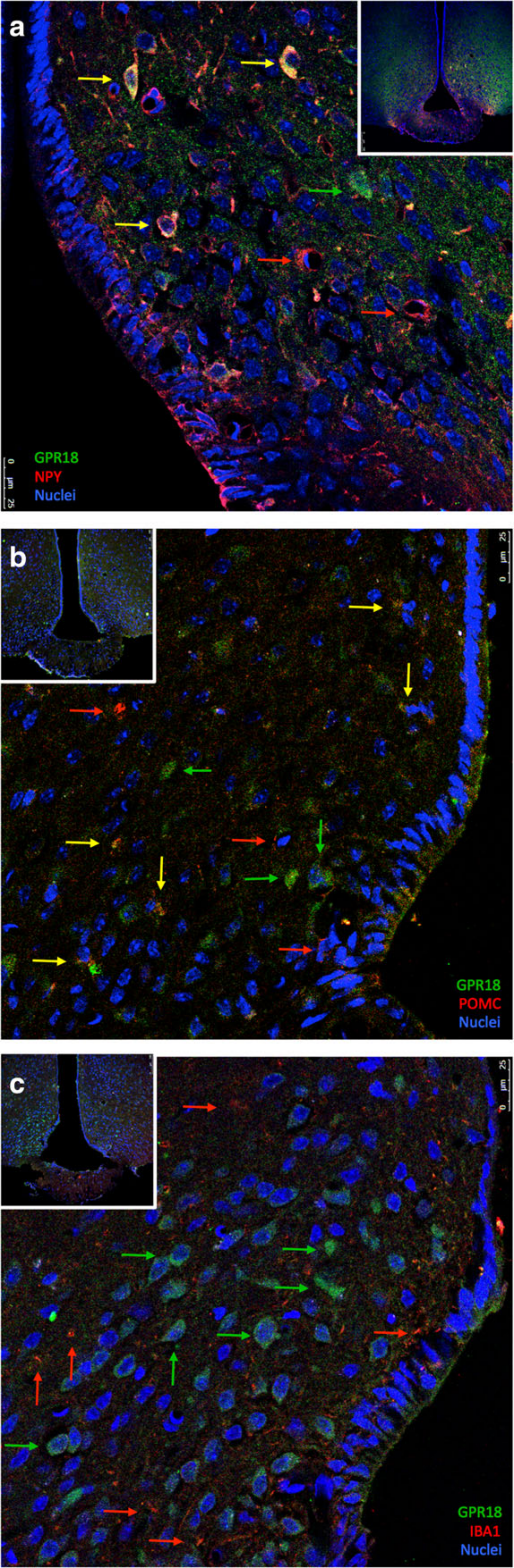
Cellular distribution of GPR18 in the hypothalamus of mice. Five-micrometre tissue sections were prepared from the hypothalamic region of lean Swiss mice and were evaluated by indirect immunofluorescence staining using antibodies against GPR18 and NPY (**a**), POMC (**b**) or Iba1 (**c**). Nuclei were stained with DAPI. *Insets* depict low magnification micrographs of the region of interest. In captions, the *colour of the arrow* represents antigens detected in respective cells and *yellow arrows* mean that both antigens are present in the respective cell. Images are representative of three independent experiments

GPR18 was recently defined as the receptor for RvD2 [27]. We employed immunofluorescence staining to determine the expression and distribution of GPR18 in the hypothalamus of lean mice. Figure 1 shows that some, but not all, of the NPY (Fig. 1a) and POMC neurons (Fig. 1b) express GPR18. However, we could not detect any Iba1-positive cells (microglia) expressing GPR18 (Fig. 1c).

### Consumption of dietary fats modulates proteins involved in RvD2 synthesis and action

**Fig. 2:**
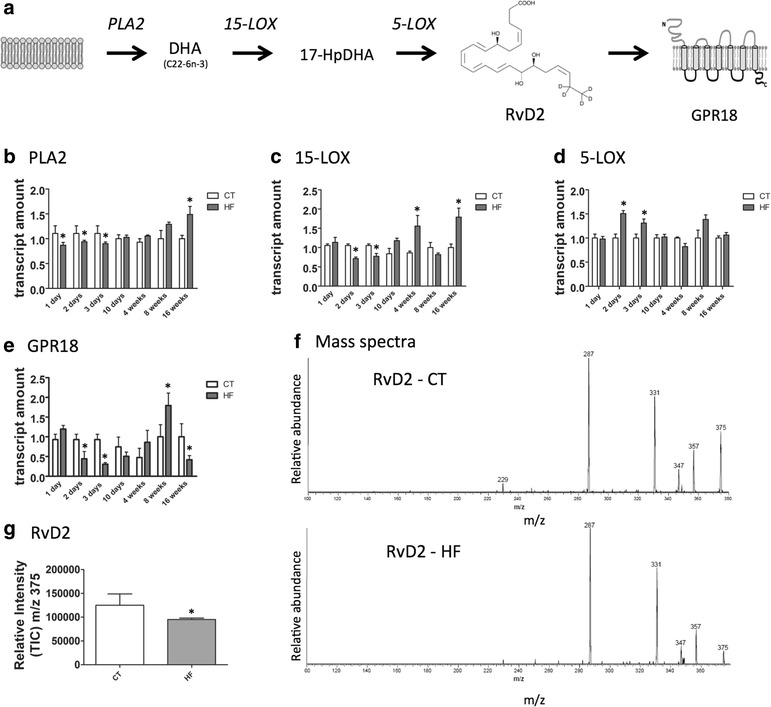
The hypothalamic expression of proteins involved in the synthesis and action of RvD2. The schematic representation of the main enzymes involved in the synthesis and the receptor for RvD2 (**a**). The transcript expressions of phospholipase A2 (**b**), 15-lipoxigenase (**c**), 5-lipoxigenase (**d**) and GPR18 (**e**) were evaluated using real-time PCR in samples collected from the hypothalamus of mice fed either chow (CT) or a high-fat diet (HF) by the time specified in the graphics (**b**–**e**). To measure hypothalamic RvD2, we employed a MALDI method, with mass spectra standard of RvD2 corresponding to m/z 375 ((**f**), *upper panel*); a sample from the hypothalamus of a mice fed on HF ((**f**), *lower panel*); quantification of RvD2 in hypothalamic samples (**g)**. In all experiments, *n* = 7. In **b**–**e** and **g**, *p < 0.05 vs. respective CT

High consumption of dietary fats is one of the most important environmental factors leading to obesity [28]. Here, mice were fed either chow or a high-fat diet (HF) that was rich in saturated fats. Then, we evaluated the hypothalamic expression of transcripts encoding for proteins involved in the synthesis and action of RvD2. In Fig. 2a, there is a schematic representation of the main enzymes involved in the synthesis of RvD2. The receptor for RvD2, GPR18 is also depicted in the scheme (Fig. 2a). PLA2 and 15-LOX, which are involved in the initial steps of RvD2 synthesis, are inhibited early after HF introduction and then undergo a significant increase at middle and late phase obesity (Fig. 2b, c). Conversely, 5-LOX, the enzyme involved in the final step of RvD2 synthesis, undergoes an early increase and then returns to levels similar to control by middle and late phase obesity (Fig. 2d). GPR18 is also regulated by dietary fats, undergoing an early reduction, then increasing at middle obesity (8 weeks) and finally reducing again at late obesity (16 weeks) (Fig. 2e).

### Hypothalamic RvD2 is reduced during late obesity

To measure hypothalamic RvD2, we employed a MALDI method, which was adapted from a mass spectrometry method, as previously described [29]. As depicted in Fig. 2f, g, RvD2, which corresponds to m/z 375, is significantly reduced in the hypothalamus of mice fed for 16 weeks on HF.

### Polyunsaturated fatty acid-rich diet increases RvD2 in the hypothalamus and improves the metabolic phenotype of obese mice

**Fig. 3:**
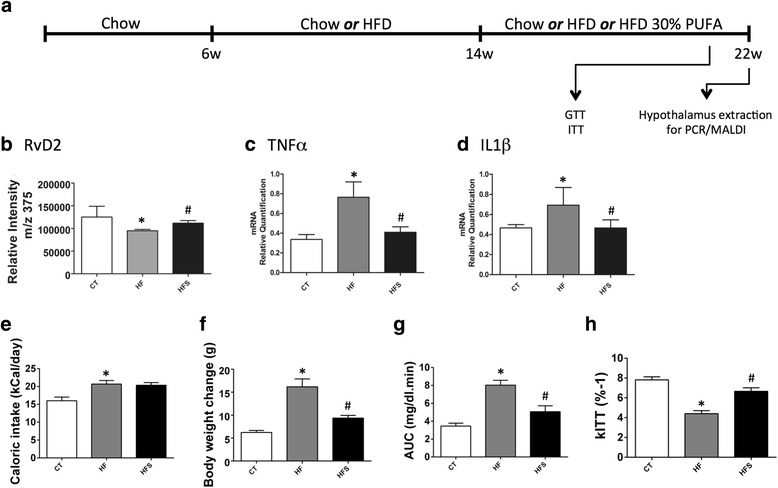
The impact of dietary substitution of saturated by unsaturated fats on the activity of the RvD2 system in the hypothalamus. Six-week-old Swiss mice were included in the study and fed on chow or HF for 16 weeks. Another group of mice was assigned to HF for 8 weeks following 8 weeks on a HF supplemented with 30% omega 3 (HFS) (**a**). Relative quantification of the RvD2 was made in the hypothalamus of mice fed either chow (CT), the high-fat diet (HF) or the HF supplemented with 30% omega 3 (HFS) (**b**). The transcript expressions of tumour necrosis factor alpha (TNFα) (**c**) and interleukin-1 beta (IL1β) (**d**) were evaluated using real-time PCR in samples collected from the hypothalamus of mice. Caloric intake (**e**) and body mass (**f**) were measured during the experimental period. At the end of the treatment, the experimental groups were subjected to a glucose tolerance test and an insulin tolerance test, and results are shown as the area under the curve (AUC) (**g**) and constant of glucose decay (kITT) (**h**). In all experiments, *n* = 7. In **b**–**h**, *p < 0.05 vs. CT and #*p* < 0.05 vs. HF

The mice were initially fed the HF diet for 8 weeks and then were randomly selected for either continuing on the current HF or transferring to a HF on which lard was substituted by a PUFA-rich oil (HFS) (Table 1); lean controls (CT) were maintained on chow throughout the experiment (Fig. 3a). PUFA substitution resulted in an increased amount of RvD2 in the hypothalamus (Fig. 3b). This was accompanied by a reduction of diet-induced expression of the pro-inflammatory cytokine transcripts TNFα (Fig. 3c) and IL1β (Fig. 3d) in the hypothalamus. In addition, the PUFA-rich diet reduced body mass gain without affecting caloric intake (Fig. 3e, f). The consumption of a PUFA-rich diet was accompanied by improved glucose tolerance, as determined via a glucose tolerance test (Fig. 3g) and by increased sensitivity to insulin, as determined by the constant of glucose decay (kITT) (Fig. 3h) during an insulin tolerance test.

### Docosahexaenoic acid induces the expression of proteins involved in the synthesis of RvD2 in the hypothalamus

**Fig. 4:**
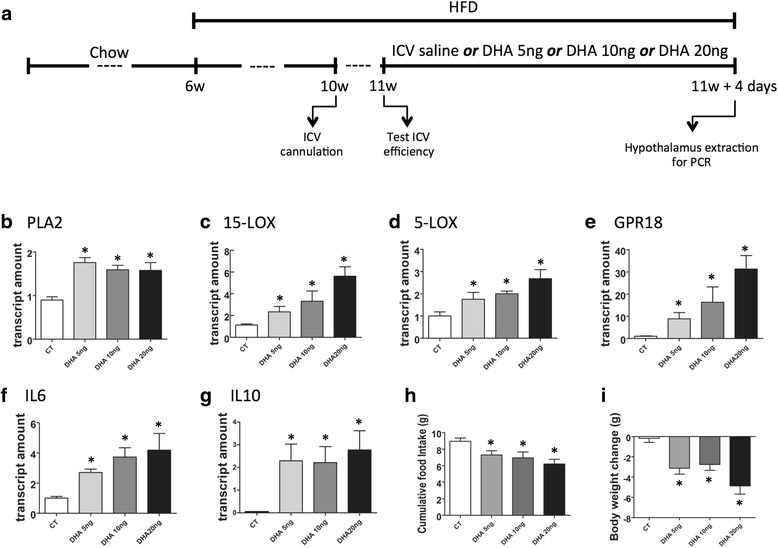
The impact of DHA-injected icv on the activity of the RvD2 system in the hypothalamus. Six-week-old Swiss mice were included in the study and fed a HF diet for 4 weeks before intracerebroventricular (icv) cannulation; after 1 week, mice were randomly selected for either saline (2 μl) or different amounts of DHA (2 μl) icv treatment for 4 days; some mice were fed on chow throughout the experimental period (CT) (**a**). At the end of the experimental period, hypothalamic RNA was extracted and employed in real-time PCR determinations of phospholipase A2 (PLA2) (**b**), 15-lipoxigenase (15-LOX) (**c**), 5-lipoxigenase (5-LOX) (**d**), GPR18 (**e**), interleukin-6 (IL6) (**f**) and interleukin-10 (IL10) (**g**). Cumulative food intake (**h**) and body mass change (**i**) were evaluated during the experimental period. In all experiments, *n* = 7. *p < 0.05 vs. CT

Next, we tested the hypothesis that injecting DHA, the substrate for RvD2 synthesis, directly in the hypothalamus could modulate the proteins involved in its synthesis. For that, mice fed the HF diet for 4 weeks were subjected to an icv cannulation and treated for 4 days, either with saline or with different amounts of DHA (Fig. 4a). As depicted in Fig. 4b–e, DHA exerted a positive effect on the expressions of PLA2, 15-LOX, 5-LOX and GPR18. In the case of 15-LOX, 5-LOX and GPR18, this was a dose-dependent effect. Because most proteins involved in the synthesis/signalling of DHA are rapidly modulated by diet (as depicted in Fig. 2b–e), we evaluated the effect of icv-injected DHA in mice fed the HF for 3 days. Additional file 1: Figure S1 shows that DHA produced increases of 15-LOX, 5-LOX and GPR18.

### Docosahexaenoic acid in the hypothalamus increases the expression of anti-inflammatory cytokines and reduces body mass

The consumption of dietary saturated fats induces hypothalamic inflammation and increases body mass [15–19]. When mice were icv-treated with DHA, there was an increased expression of two anti-inflammatory cytokines in the hypothalamus—IL6 (Fig. 4f) and IL10 (Fig. 4g)—and no modification of the expression of TNFα and IL1β (not shown). This was accompanied by reduced caloric intake (Fig. 4h) and reduced body mass (Fig. 4i). DHA was also capable of inducing the increased hypothalamic expression of IL6 and IL10 in mice fed the HF for 3 days (Additional file 1: Figure S1).

### RvD2 improves inflammatory and metabolic phenotypes in obese mice

**Fig. 5:**
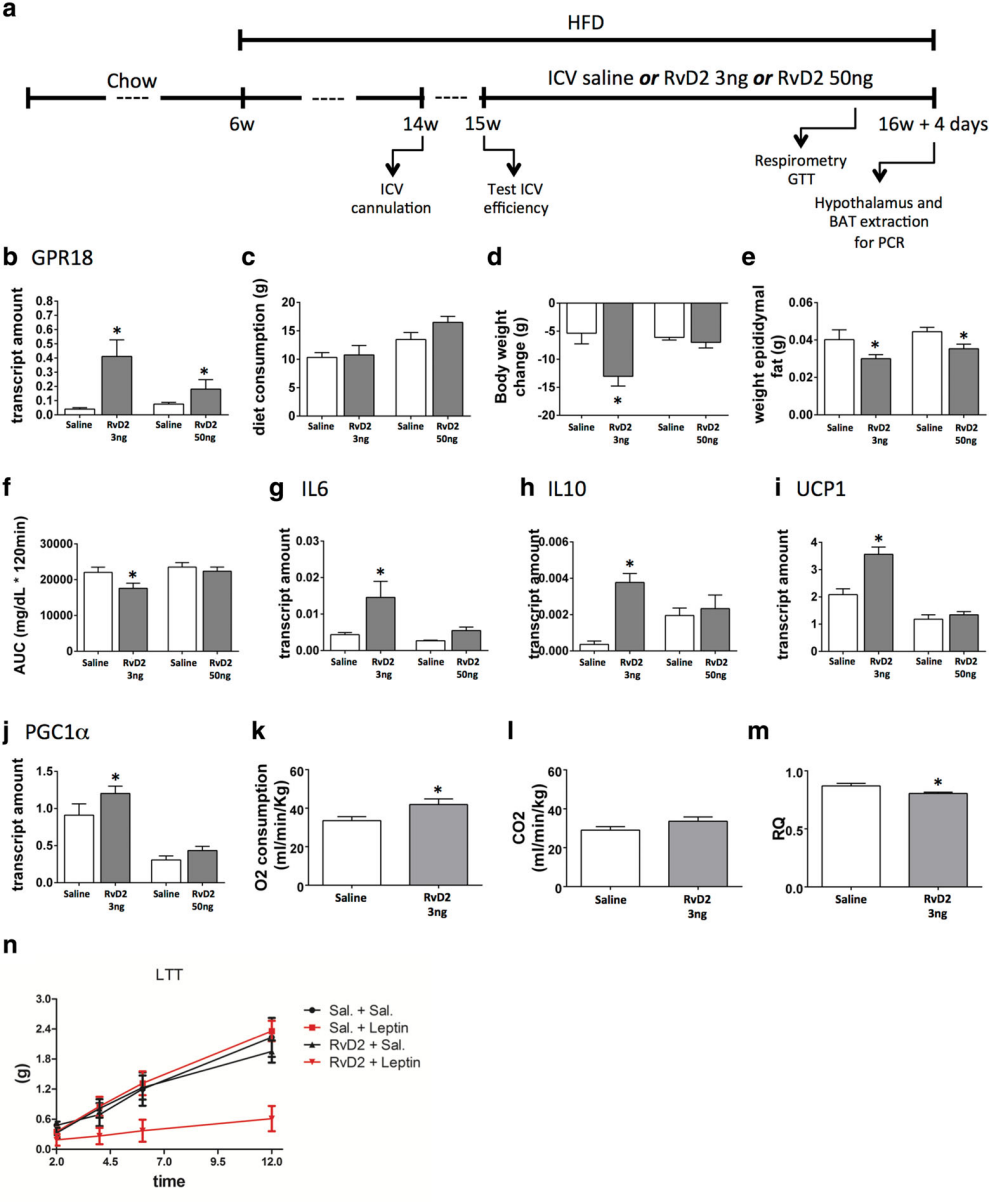
The impact of exogenous RvD2-injected icv on inflammatory and metabolic parameters in mice. Six-week-old Swiss mice were included in the study and fed a HF diet for 8 weeks before intracerebroventricular (icv) cannulation; after 1 week, mice were randomly selected for either saline (2 μl) or different amounts of RvD2 (2, 3 or 50 ng) icv treatment for 11 days (**a**). At the end of the experimental period, hypothalamic RNA was extracted and employed in real-time PCR determinations of GPR18 (**b**). Cumulative food intake (**c**) and body mass variation (**d**) were determined during the experimental period. At the end of the experimental period, the epididymal fat pad was measured (**e**). In addition, mice were submitted a glucose tolerance test, and results are expressed as the area under the curve (AUC) (**f**). Interleukin-6 (IL6) (**g**) and interleukin-10 (IL10) (**h**) transcripts were determined in samples from the hypothalamus, whereas uncoupling protein 1 (UCP1) (**i**) and PGC1a (**j**) transcripts were determined in samples from the brown adipose tissue. Some mice were subjected to indirect calorimetry, resulting in the values for O_2_ consumption (**k**), CO_2_ production (**l**) and respiratory quotient (**m**). In addition, some mice were subjected to a leptin tolerance test (LTT) and results are expressed as cumulative food intake during 12 h (**n**). In the experiments reported in panels **a**–**j**, *n* = 8; in the experiments reported in panels **k**–**n**, *n* = 5. *p < 0.05 vs. saline. Experiments for evaluation of 3 and 50 ng RvD2 in the hypothalamus were performed in different occasions. The differences obtained in some of the saline groups reflect interexperimental variability. For statistical analysis, group-specific saline control was considered as baseline

Obese mice were submitted to icv cannulation and treated with RvD2 icv for 11 days (Fig. 5a). Either 3.0 or 50 ng RvD2 was sufficient to increase the hypothalamic expression of GPR18 (Fig. 5b). RvD2 icv was not capable of modifying caloric intake (Fig. 5c); nevertheless, 3.0 ng icv was sufficient to reduce body mass gain (Fig. 5d), while both 3.0 and 50 ng produced a significant reduction of visceral fat (Fig. 5e). The 3.0 ng, but not the 50 ng, dose improved glucose tolerance, as determined by the area under the glucose curve during a GTT (Fig. 5f). The 3.0 ng, but not the 50 ng, dose led to an increased expression of the anti-inflammatory cytokines IL6 (Fig. 5g) and IL10 (Fig. 5h) and no modification of the expression of TNFα and IL1β (not shown) in the hypothalamus. In addition, 3.0 ng RvD2-injected icv increased the expressions of UCP1 (Fig. 5i) and PGC1α (Fig. 5j) in the BAT. This was accompanied by the increased whole body consumption of O_2_ (Fig. 5k) and reduced respiratory quotient (Fig. 5m). Resistance to leptin is a hallmark of diet-induced obesity [30]. In the final part of this study, we tested the hypothesis that RvD2 could revert hypothalamic leptin resistance in obese mice. For that, obese mice were initially treated icv with saline or RvD2 and then treated with either saline or leptin. Spontaneous intake of diet was measured over a period of 12 h. As depicted in Fig. 5n, RvD2 was sufficient to significantly reduce food intake in response to leptin.

## Discussion

In experimental obesity, the consumption of dietary fats leads to the rapid activation of an inflammatory response in the hypothalamus [20]. Over time, neurons of the medium-basal hypothalamus involved in the control of food intake and energy expenditure are affected by inflammation and become dysfunctional [15–19]. The anomalous activity of such neurons is characterised, at least in part, by their reduced responsiveness to the adipostatic hormones, leptin and insulin [18, 31, 32]. In addition, upon prolonged exposure to dietary fats, more dramatic outcomes may occur, such as defects in mitochondrial function [33, 34], anomalous regulation of autophagy [35, 36] and the ubiquitin/proteasome system [21] and, eventually, increased neuronal apoptosis [15].

Several approaches employing genetic and pharmacological tools to dampen hypothalamic inflammation were successful in reducing adiposity and improving the metabolic phenotypes associated with obesity [19, 37]. More recently, reduction of diet-induced hypothalamic inflammation and improvement of the obese phenotype were obtained by the use of DHA, either in the diet or directly injected into the hypothalamus [23]. Since DHA is the substrate for RvD2 synthesis, we decided to evaluate if the hypothalamus is equipped for producing this resolvin, and if so, how would it be regulated in diet-induced obesity.

Initially, using immunofluorescence, we showed that GPR18, the receptor for RvD2, is expressed in the hypothalamus, particularly in POMC and NPY neurons. We could not detect any labelling for GPR18 in the microglia. No previous study has evaluated the expression and distribution of GPR18 in the brain. However, at least one study has used RvD2 to treat chronic pain by injecting the substance into the spine, which suggests that sensory neurons are responsive to this resolvin and may express GPR18 [38].

Next, we evaluated the hypothalamic expression of enzymes involved in the conversion of DHA to RvD2. Both in lean and obese animals, we detected the presence of all components of the RvD2 synthetic pathway and its receptor. Interestingly, upon high-fat feeding, there was a modulation of expression of all elements of the synthetic pathway and the receptor. PLA2 and 15-LOX were initially inhibited during the first days after beginning the diet and then underwent an increase at late stage obesity. Conversely, 5-LOX was initially stimulated and then normalised. We also measured the amount of RvD2, which was reduced at late stage obesity. A study has shown that exogenous 17-HpDHA reduces inflammation and attenuates insulin resistance in an animal model of obesity [13]. This could suggest that, in our model, the increased PLA2 and 15-LOX, accompanied by baseline levels of 5-LOX, would intuitively result in the accumulation of endogenous 17-HpDHA. However, rather than activating an anti-inflammatory response, we see an increase of pro-inflammatory markers. This is most possibly due to the fact that both RvD2 and its receptor GPR18 are reduced.

There is very limited information about the production and function of RvD2 in the brain. In a study aimed at developing methods for the detection and measurement of resolvins, the presence of RvD2 was detected in the brain of mice subjected to a stroke, suggesting that it could play a role in attenuation of the ischemic lesion [29]. Another study evaluated the post-mortem brain of patients with Alzheimer’s disease (AD) [39]. Interestingly, there was a direct correlation between the cerebrospinal fluid levels of RvD2 and the cognitive scores of the patients. The authors suggested that a defect in the production of resolvins in the brain could be connected to the evolution of AD. Similarly, an experimental study has demonstrated that ageing rats treated with DHA present improved memory, which was accompanied by increased brain levels of RvD2 [40].

Besides its role as an endogenous substance produced to control the magnitude and duration of inflammation, much attention has been devoted to resolvins because of their potential use as exogenously delivered therapeutic agents [6]. Some studies have employed DHA or other substrates to induce the synthesis of endogenous RvD2 [13, 40], whereas others have used RvD2 directly [38]. Here, we first evaluated the impact of dietary substitution of saturated by unsaturated fats on the activity of the RvD2 system in the hypothalamus; next, we treated mice with DHA-injected icv in the hypothalamus. Last, we treated mice with RvD2 icv in the hypothalamus. All three approaches were very consistent to improve the obesity-associated metabolic phenotype of mice. In addition, both DHA and exogenous RvD2 were capable of inducing the hypothalamic expression of two cytokines with anti-inflammatory activity in the brain, IL10 and IL6. It is worthwhile to mention that, in the case of direct injection of RvD2 in the hypothalamus, the lower dose, 3.0 ng, provided better results than 50 ng. Although we did not explore this difference in detail, in other context, a large dose of resolvin resulted in a less robust anti-inflammatory action, suggesting the existence of a dose-dependent desensitizing effect for this class of substances [41].

It has been shown that at least some of the beneficial effects of physical activity in the control of food intake and body adiposity are due to the increased expression of IL10 and IL6 in the hypothalamus [42]. In fact, IL10 can attenuate not only mild inflammatory activity in the hypothalamus, as the one associated with obesity [42], but also more severe inflammatory activity, such as the one induced by LPS [43]. Likewise, icv injection of exogenous IL6 can reduce food intake and body mass [44], whereas endogenous IL6 can mediate some of the beneficial effects of GLP1, reducing food intake and body mass [45]. Thus, at least part of the effects of RvD2 in the hypothalamus may be mediated by the increased expression of IL6 and IL10.

An important aspect of the beneficial effects of RvD2 in the hypothalamus is its capacity to improve glucose tolerance in obese mice. Although this effect could be due to body mass reduction, recent studies have shown that the simple attenuation of diet-induced hypothalamic inflammation can, through neural connections, reduce hepatic glucose output [46] while increasing insulin production [47]. Testing this hypothesis was not a goal of the present study; however, as glucose tolerance improved with short-term use of RvD2 (11 days) and even in the presence of a mild body mass reduction, we propose that at least part of the effect could be due to neural mechanisms. This is further supported by the fact that, upon RvD2 treatment, there were increased expressions of UCP1 and PGC1α in the BAT, which is known to be mediated by sympathetic inputs [48].

## Conclusions

This is the first study demonstrating the presence of RvD2 in the central nervous system, particularly in the hypothalamus. Both endogenously produced and exogenously administered RvD2 improved the obese and metabolic phenotypes of mice fed a high-fat diet. Thus, RvD2 and other resolvins may be attractive approaches to reduce obesity-associated hypothalamic inflammation.

## Additional file

Additional file 1: Figure S1. Six-week-old, male Swiss mice were stereotaxically instrumented in a Stoelting stereotaxic apparatus to receive a cannula placed in the lateral hypothalamic ventricle, using the following stereotaxic coordinates: anteroposterior 0.34 mm, lateral 1.0 mm, and dorsoventral 2.2 mm. The correct position of the cannula was tested 5 days after surgery by evaluation of the thirst response elicited by intracerebroventricular (icv) angiotensin II (10^6^ M). After 1 week, icv-cannulated mice were transferred to the high-fat diet and treated once a day for 3 days with 2 μL of saline (CTR) or 2 μL of DHA (5, 10 or 20 ng). Caloric intake and body mass were determined during the experimental period. At the end of the experimental period, the hypothalamus was obtained for real-time PCR quantitative determination of the transcripts encoding for PLA2 (A), 15-LOX (B), 5-LOX (C), GPR18 (D), IL6 (E) and IL10 (F). The methods for hypothalamic extraction and real-time PCR are described in the Methods section of the paper. (PDF 768 kb)

## Abbreviations

AD: Alzheimer’s disease
ANOVA: Analysis of variance
DHA: Docosahexaenoic acid
GAPD: Glyceraldehyde-3-phosphate dehydrogenase
GLP1: Glucagon-like peptide 1
GPR: G-protein-coupled receptor
GTT: Glucose tolerance test
HDHA: Hydroxydocosahexaenoic acid
HF: High fat
HFS: High fat substituted
IL10: Interleukin 10
IL1β: Interleukin 1 beta
IL6: Interleukin 6
ITT: Insulin tolerance test
LOX: Lipoxygenase
LPS: Lipopolysaccharide
LTT: Leptin tolerance test
NPY: Neuropeptide Y
PGC1a: Peroxisome proliferator-activated receptor gamma coativator 1 alpha
PLA: Phospholipase
POMC: Proopiomelanocortin
PUFA: Polyunsaturated fatty acid
RvD1: Resolvin D1
RvD2: Resolvin D2
TNFα: Tumour necrosis factor alpha
UCP1: Uncoupling protein 1
