# Supplementary material for: Resolvin RvD2 reduces hypothalamic inflammation and rescues mice from diet-induced obesity
Source: J Neuroinflammation. 2017 Jan 5;14:5. doi: 10.1186/s12974-016-0777-2 (PMC5234140; doi:10.1186/s12974-016-0777-2)

Pascoal et al.

Resolvin RvD2 reduces hypothalamic inflammation and rescues mice from diet-induced obesity

Additional file 1: Figure S1

Six-week old, male Swiss mice were stereotaxically instrumented in a Stoelting stereotaxic apparatus to receive a cannula placed in the lateral hypothalamic ventricle, using the following stereotaxic coordinates: antero-posterior: 0.34 mm; lateral: 1.0 mm; dorso-ventral: 2.2 mm. The correct position of the cannula was tested five days after surgery by evaluation of the thirst response elicited by intracerebroventricular (icv) angiotensin II (10^{6}M). After one week, icv cannulated mice were transferred to the high-fat diet and treated once a day for three days with 2µL of saline (CTR) or 2µL of DHA (5ng, 10ng or 20ng). Caloric intake and body mass were determined during the experimental period. At the end of the experimental period, the hypothalamus was obtained for real-time PCR quantitative determination of the transcripts encoding for PLA2 (A), 15-LOX (B), 5-LOX (C), GPR18 (D), IL6 (E) and IL10 (F). The methods for hypothalamic extraction and real-time PCR are described in the Methods section of the paper.

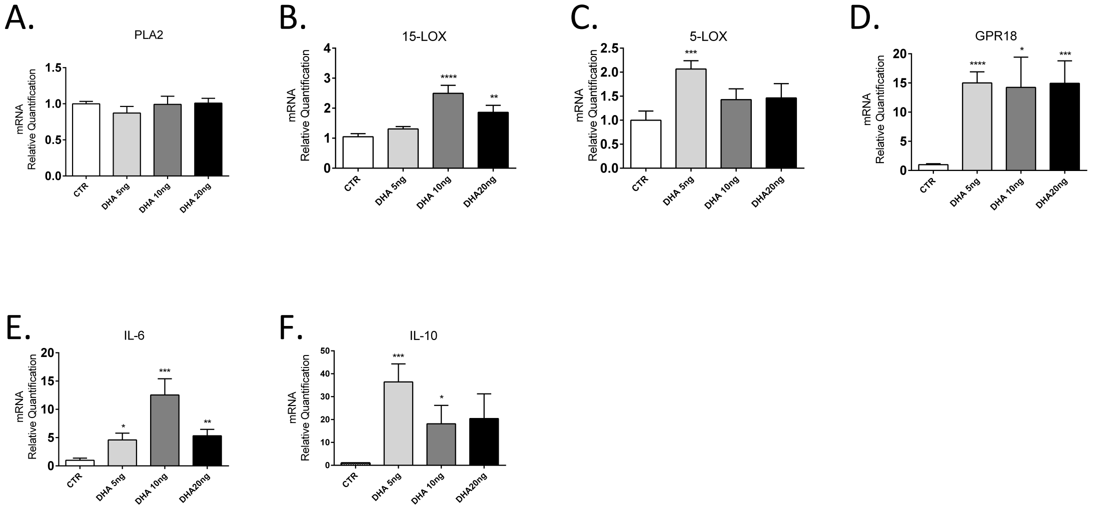

Supplement: Additional file 1: Figure S1. — Six-week-old, male Swiss mice were stereotaxically instrumented in a Stoelting stereotaxic apparatus to receive a cannula placed in the lateral hypothalamic ventricle, using the following stereotaxic coordinates: anteroposterior 0.34 mm, lateral 1.0 mm, and dorsoventral 2.2 mm. The correct position of the cannula was tested 5 days after surgery by evaluation of the thirst response elicited by intracerebroventricular (icv) angiotensin II (106 M). After 1 week, icv-cannulated mice were transferred to the high-fat diet and treated once a day for 3 days with 2 μL of saline (CTR) or 2 μL of DHA (5, 10 or 20 ng). Caloric intake and body mass were determined during the experimental period. At the end of the experimental period, the hypothalamus was obtained for real-time PCR quantitative determination of the transcripts encoding for PLA2 (A), 15-LOX (B), 5-LOX (C), GPR18 (D), IL6 (E) and IL10 (F). The methods for hypothalamic extraction and real-time PCR are described in the Methods section of the paper. (PDF 768 kb) [file 12974_2016_777_MOESM1_ESM.pdf]
